# The Anti-Biofilm Properties of Phloretin and Its Analogs against *Porphyromonas gingivalis* and Its Complex Flora

**DOI:** 10.3390/foods13131994

**Published:** 2024-06-24

**Authors:** Desheng Wu, Lisha Hao, Xiaohan Liu, Xiaofeng Li, Guanglei Zhao

**Affiliations:** 1 School of Food Science and Engineering, South China University of Technology, Wushan Road 381, Guangzhou 510640, China; 202010105392@mail.scut.edu.cn (D.W.); 202220126744@mail.scut.edu.cn (L.H.); felxh6005@mail.scut.edu.cn (X.L.); 2 State Key Laboratory of Pulp and Paper Engineering, South China University of Technology, Guangzhou 510641, China

**Keywords:** *Porphyromonas gingivalis*, biofilm, polyphenols, flavonoids, structure–activity relationships

## Abstract

*Porphyromonas gingivalis* is crucial for the pathogenesis of periodontitis. This research investigated the effects of the fruit-derived flavonoid phloretin and its analogs on the growth of pure *P. gingivalis* and the flora of *P. gingivalis* mixed with the symbiotic oral pathogens *Fusobacterium nucleatum* and *Streptococcus mitis*. The results showed that the tested flavonoids had little effect on the biofilm amount of pure *P. gingivalis*, but significantly reduced the biofilm amount of mixed flora to 83.6~89.1%. Biofilm viability decreased to 86.7~92.8% in both the pure- and mixed-bacterial groups after naringenin and phloretin treatments. SEM showed that phloretin and phlorizin displayed a similar and remarkable destructive effect on *P. gingivalis* and the mixed biofilms. Transcriptome analysis confirmed that biofilm formation was inhibited by these flavonoids, and phloretin significantly regulated the transcription of quorum sensing. Phlorizin and phloretin reduced AI-2 activity to 45.9% and 55.4%, respectively, independent of the regulation of related gene transcription. This research marks the first finding that these flavonoids possess anti-biofilm properties against *P. gingivalis* and its intricate bacterial community, and the observed performance variations, driven by structural differences, underscore the existence of intriguing structure–activity relationships.

## 1. Introduction

Numerous studies over several decades have established a direct link between the Gram-negative bacterium *Porphyromonas gingivalis* and periodontal disease. Globally, a significant portion of the population is affected by periodontal disease, with the overall prevalence of periodontitis exceeding 40% and the global age-standardized prevalence of severe periodontal disease standing at 11.2% [1,2]. Periodontitis, associated with tooth loss, is linked to systemic diseases such as cardiovascular disease, rheumatoid arthritis, and Alzheimer’s disease [3]. Periodontal disease is not a single-factor infectious disease, but rather, is caused by the collaboration of various microorganisms that form a pathogenic entity when the homeostasis of the oral microbiota is disrupted [4]. When homeostasis is disrupted, *P. gingivalis* will exert a significant influence, further interfering with the homeostasis of oral flora and promoting the development of periodontal disease. While *P. gingivalis* itself can exert many pathogenic effects, other oral microorganisms, such as *Fusobacterium nucleatum* and *Streptococci*, also play a crucial role in supporting *P. gingivalis* [5,6].

The cooperation of *P. gingivalis* with other microorganisms has led to the establishment of a symbiotic relationship between *P. gingivalis* and certain microorganisms, and the formation of multi-species biofilms has created a more favorable living environment for *P. gingivalis* [7,8]. In this symbiotic relationship, *F. nucleatum* and *Streptococci* play pivotal roles. In the oral cavity, *Streptococci*, *Actinomyces*, and *Veillonellae* adhere to the tooth surface, serving as a biofilm substrate to which other bacteria can bridge, thereby resulting in coaggregation [9]. *Streptococci* produce various adhesins that bind to the salivary membrane and other oral bacteria, enhancing signaling between the microbiota [10]. *F. nucleatum* colonizers can coaggregate at all stages, making it one of the most critical bridge strains. *F. nucleatum* coaggregates with *P. gingivalis*, and both species coaggregate with *S. gordonii*; this combination creates a stable multi-species biofilm, providing a solid foundation for the performance of *P. gingivalis* [11,12]. *F. nucleatum* plays a protective role in the oral biofilm, allowing *P. gingivalis* to grow in an unfavorable oxygen-containing environment by shaping a microenvironment with reduced oxygen tension [13].

In general, while *P. gingivalis* serves as the commander of the oral microbiota’s attack on the human body, it also relies on the assistance of other microorganisms. *P. gingivalis* itself lacks strength in generating stable biofilms and has poor resistance to oxygenated environments. Collaboration with *Streptococci*, *F. nucleatum*, and other bacteria effectively compensates for these shortcomings and significantly enhances the threat posed by *P. gingivalis*. Disruption of this collaboration would help address issues related to *P. gingivalis*.

The treatment of *P. gingivalis* traditionally uses physical scaling treatment (e.g., tooth scaling) and antibiotics such as amoxicillin, doxycycline, and metronidazole. However, when a biofilm is formed under the gums, the effectiveness of antibiotic treatment will be greatly reduced, thus requiring higher dosages to be used. For example, doxycycline requires an 8–64 times higher dosage to reproduce the minimum bactericidal concentration, and for amoxicillin and metronidazole, they require 2–8 times higher doses to treat the biofilm compared to planktonic cells [14]. This exacerbates the problems of the side-effects of these medicines and increasing bacterial resistance.

To solve similar problems, lots of research has been focusing on plant-derived natural products due to their safety, unique structures, and the abundance [15]. Many natural products have been proven to have antibacterial effects, but only a small number of them have been reported to inhibit *P. gingivalis* biofilms or inhibit coaggregation [16,17]. Moreover, most of these experiments do not clearly explain the specific mechanism of action, or just use mixed extracts for their research, or the chemical structure of the natural products is not elucidated. Therefore, it is necessary to select some representative compounds, conduct in-depth research on their structure–activity relationships, and explore their potential.

Polyphenols are commonly found in many types of human diets and have been found to have significant direct and indirect benefits on oral health, with great potential in preventing and treating dental problems [18]. According to existing research reports, a variety of polyphenols, including tea polyphenols (mainly catechins, from tea), berry polyphenols (mainly proanthocyanidins, from berry and grape), resveratrol (from grape and *Polygonum cuspidatum*), etc., have been reported to have the effect of inhibiting *P. gingivalis* or alleviating post-infection symptoms [19,20,21,22]. Most of these polyphenols have a flavonoid structure. Among them, potential structure–activity relationships have been reported for chalcones in inhibiting several oral pathogenic bacteria, including differences in antibacterial properties and changes in anti-inflammatory capabilities [23,24]. However, there are no reports exploring the effect of the structure–activity relationship on the ability of chalcones to inhibit *P. gingivalis* biofilms.

In our previous work, phloretin was screened for its ability to inhibit the virulence of *P. gingivalis* and had a potential structure–activity relationship, but it was not clear whether it had the ability to inhibit biofilm activity. Phloretin is a chalcone flavonoid widely distributed in apple fruits, bark, and leaves, and has multiple biological functions [25]. Phloretin and its analog phlorizin can regulate the metabolism of apple trees and perform an antibacterial role, and phloretin performs better than phlorizin in most antibacterial tests. Studies have reported the antibacterial ability of phloretin against *Staphylococcus aureus*, *Serratia marcescens*, and other bacteria and its interference with biofilm formation, and phloretin has the ability to inhibit the biofilm formation of the oral pathogenic bacteria *Streptococcus mutans*, and has the ability to inhibit its quorum-sensing ability [26,27,28]. However, there are few studies on the application of phloretin and phlorizin to *P. gingivalis*, and only one study has reported the effects of total apple polyphenols on inhibiting *P. gingivalis* [29].

Although the specific mechanism of total apple polyphenols is not yet clear, and the specific active ingredients have not been identified by the research, their effectiveness has been preliminarily confirmed, and this can be used as a breakthrough point for in-depth research to clarify whether there is a structure-activity relationship effect when they are inhibiting *P. gingivalis*. Therefore, it is necessary to select structural analogs for structure–activity relationship analysis. Using phloretin as a benchmark reference, structural analogs were searched to explore the impact of structural changes on its performance.

**Figure 1:**
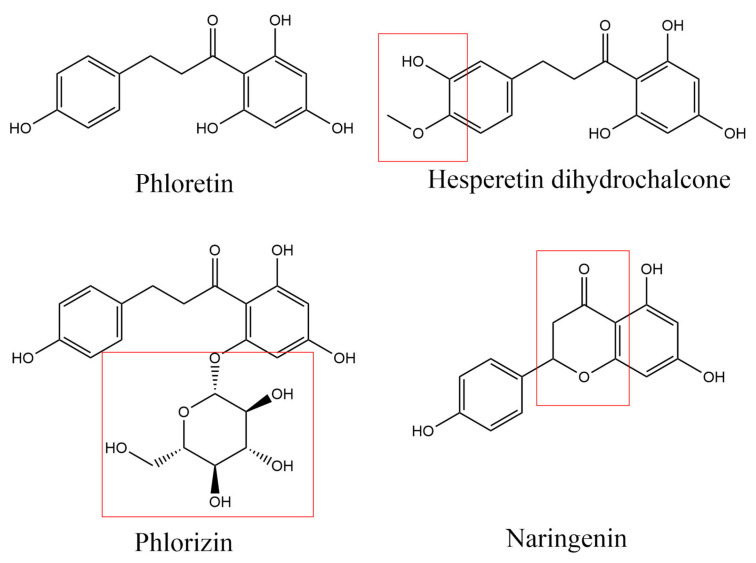
Molecular structures of phlorizin, naringenin, hesperetin dihydrochalcone, and phloretin. The regions of each molecule that differ in chemical structure from phloretin are marked.

Phlorizin, naringenin, hesperetin dihydrochalcone, and phloretin are a group of flavonoids with similar structures (Figure 1). Phlorizin is a derivative of phloretin, which has an additional glycoside in its structure and is also widely distributed in apples; it may play a similar role to phloretin in the application of total polyphenols in apples. Naringenin mainly exists in edible fruits such as citrus fruits and tomatoes [30]. It is hydrolyzed from naringin and has a wide range of biological effects. In addition, some studies have reported that naringenin can inhibit the growth of *P. gingivalis*, *F. nucleatum*, and *S. mitis*, but there are no reports related to *P. gingivalis* biofilms [22]. Naringenin has a similar structure to phloretin except that it does not have a dihydrochalcone structure, and it can be used to determine the significance of the dihydrochalcone structure on the function of phloretin. Hesperetin dihydrochalcone is a chalcone compound with a similar structure to phloretin, and serves as a comparative example for other dihydrochalcones. It is a derivative of neohesperidin dihydrochalcone, and is mainly used for taste regulation [31]. There are very few reports on its biological activity.

Through these structural analogs, the effects of adding glycosides, ring closing, and methoxy groups on the performance of phloretin in inhibiting *P. gingivalis* can be explored. There are few studies related to the inhibition of *P. gingivalis* by these flavonoids, and moreover, no study has yet analyzed the effects of these candidate flavonoids on *P. gingivalis biofilm* formation at the transcriptome level. Research in this field will help expand the application value of these food-borne ingredients.

In summary, based on preliminary research work, four flavonoids, phlorizin and its structural analogs phloretin, naringenin, and hesperetin dihydrochalcone, were selected in this experiment to explore their structure-activity relationships and their contribution to solving the problems related to *P. gingivalis*. The presence of *P. gingivalis* alone and in cooperation with *F. nucleatum* and *S. mitis* was considered and studied. Bacterial morphological changes and biofilm structure were explored with scanning electron microscopy (SEM). The activity of autoinducer-2 (AI-2) was also measured to assess interbacterial communication. The effects of candidate flavonoids on gene expression in *P. gingivalis* were also confirmed by transcriptomics. The results show that subtle changes in structure have a significant impact on the effectiveness of candidate molecules. This information on structure–activity relationships will help guide the screening and transformation of lead compounds in subsequent research.

## 2. Materials and Methods

### 2.1. Reagents and Chemicals

BHI broth and BHI agar were purchased from Hope Bio-Technology Co., Ltd. (Qingdao, China). Defibrinated sheep blood was obtained from Hongquan Bio Co., Ltd. (Guangzhou, China). Bovine hemoglobin and resazurin were obtained from Yuanye Bio-Technology Co., Ltd. (Shanghai, China). Phlorizin, phloretin, and naringenin were obtained from Rhawn Reagent Co., Ltd. (Shanghai, China). Hesperetin dihydrochalcone was obtained from Zzstandard Co., Ltd. (Shanghai, China). The purity of the compounds used in the experiment was not less than 98%. PBS, methanol, ethanol, glacial acetic acid, xylene, and crystal violet were purchased from Sigma-Aldrich Co., Ltd. (Darmstadt, Germany). Glutaraldehyde fixative was obtained from Bioisco Co., Ltd. (Lianyungang, China).

### 2.2. Preparation of Bacterial Culture

*Porphyromonas gingivalis* W83 (ATCC BAA-308), *Fusobacterium nucleatum* (ATCC 25586), and *Streptococcus mitis* (ATCC 49456) strains were purchased from the Guangdong Microbial Culture Collection Center (Guangzhou, China). The sheep blood was centrifuged at 400× *g* for 20 min with a H1750R high-speed refrigerated centrifuge (Cence Co., Ltd., Changsha, China), the serum was collected, and 3% sheep blood serum and 100 mg/L bovine hemoglobin were added to the sterilized BHI medium. All the strains were inoculated into the prepared medium and incubated in an anaerobic incubator (MGC Co., Ltd., Tokyo, Japan) at 37 °C for 24 h. For transcriptome sequencing, the bacteria were incubated for 48 h.

### 2.3. Preparation of Biofilm

BS-18-RC cell sheets (Biosharp Co., Ltd., Hefei, China) were added to a sterilized 12-well plate filled with bacterial culture, as a substrate to allow adhesion and biofilm formation. For the *P. gingivalis* biofilm experiment, 1.8 mL of bacterial culture was added to the well plate, and then incubated in an anaerobic incubator at 37 °C for 96 h. We added 200 μL 1 mg/mL candidate drug solution to the well plate to make the final concentration of the drug 100 μg/mL, and continued to incubate for 24 h to prepare for subsequent experiments. In the blank group, only distilled water was added.

For the mixed-bacterial biofilm experiments, we added 0.5 mL each of *F. nucleatum* and *S. mitis* bacterial liquid to the well plate, incubated it in an anaerobic incubator at 37 °C for 24 h, and then added 0.8 mL of *P. gingivalis* bacterial liquid, and continue to incubate for 72 h. We added 200 μL 1 mg/mL candidate drug solution to the well plate to make the final concentration of the drug 100 μg/mL, and continued to incubate for 24 h to prepare for subsequent experiments. In the blank group, only distilled water was added.

The metabolic activity of the biofilm was detected based on the ability of cells to reduce resazurin [32]. We took the biofilm prepared in the above step (surface-attached cells), added fresh culture medium, and then added 5% (*v*/*v*) 100 mg/mL resazurin solution, and incubated it at 37 °C. We took a sample and monitored its fluorescence intensity at 570/590 nm for 180 min using a microplate reader. The percentage change in biofilm viability was calculated based on fluorescence intensity. The obtained data were normalized based on the blank group.

### 2.4. Biofilm Staining

We took the well plate with the prepared biofilm, removed the planktonic cells and culture medium, and washed the wells with sterile physiological saline. We added 1 mL of methanol to each well, fixed it for 15 min, removed the methanol, and let it dry naturally for 15 min. We added 1 mL of 1% crystal violet solution to each well. After staining at 25 °C for 10 min, we removed the staining solution and washed away the excess dye with sterile PBS. We dried the well plate at 37 °C, and then add 1 mL of 33% glacial acetic acid solution to each well, and incubated it at 37 °C for 30 min for decolorization. We used a microplate reader to check the absorbance value at 550 nm and calculated the biofilm content. We assumed that the absorbance value of the blank group was 100%, and calculated the relative content of biofilm in the other groups. The obtained data were normalized based on the blank group.

### 2.5. Autoinducer-2 Detection

*Vibrio harveyi* is the bacterium with the most obvious characteristics of AI-2 signaling. AI-2 can induce its luminescence, and the luminescence intensity is positively related to the concentration of AI-2 [33].

Candidate flavonoids were added to the *P. gingivalis* bacterial solution and make the final concentration of the drug 100 μg/mL. In the blank group, only distilled water was added. The bacterial solution was incubated anaerobically at 37 °C for 12 h, and then centrifuged at 10,000× *g* for 10 min to separate the supernatant. The biosensor *V. harveyi* BB170 was obtained from the BeNa Culture Collection (Zhengzhou, China). The strain was grown overnight in 2216E medium at 30 °C and shaken at 100 r/min, and then diluted in a ratio of 1:2000 into fresh 2216E medium. Then, 180 μL of diluted *V. harveyi* bacterial solution and 20 μL of *P. gingivalis* supernatant were added to a 96-well plate, mixed, and incubated at 30 °C in the dark for 5 h. We used a microplate reader to detect the chemiluminescence of *V. harveyi* at Ex485/Em528 nm at 0 h and 5 h, and obtained the AI-2 signal intensity. The obtained data were normalized based on the blank group.

### 2.6. Scanning Electron Microscopy

We took the prepared well plate with the bacteria, washed it three times with PBS, added 2 mL of 2.5% pentylene glycol fixative (for electron microscopy), and kept it overnight at 4 °C. After removing the fixative, gradient elution was performed with ethanol, with an elution time of 15 min, and finally, we air-dried the samples. The samples were sputtered with gold, and then loaded and photographed according to the instrument operating procedures. In this experiment, the SEM equipment used for photography included a JSM-7900F (JEOL Co., Ltd., Tokyo, Japan) and an EVO 18 (Carl Zeiss Co., Ltd., Oberkochen, Deutschland).

### 2.7. Transcriptome Sequencing

We took the bacterial culture for RNA sequencing prepared in step 2.2 and added candidate flavonoids to a final concentration of 100 μg/mL. We incubated the culture solution anaerobically at 37 °C for 4 h, and then centrifuge it at 6000× *g* for 20 min and removed the supernatant. The precipitate cells were quickly frozen in liquid nitrogen and stored at −80 °C for RNA extraction. For all samples, this process was repeated twice.

Total RNA was extracted using a TRIzol-based method (Life Technologies, Carlsbad, CA, USA) or a HiPure Universal RNA Mini Kit (Magen, Guangzhou, China). The rRNA was removed from the total RNA, and we purified the sample with RNA Clean XP Beads (AGENCOURT, Brea, CA, USA). Then, the mRNA was fragmented into 200 nt and purified with RNA Clean XP Beads. First-strand and second-strand cDNA were synthesized by a reaction kit (New England BioLabs, Ipswich, MA, USA). Then, the cDNA/DNA/Small RNA libraries were sequenced on an Illumina sequencing platform by Genedenovo Biotechnology Co., Ltd. (Guangzhou, China) with a read length of 150 bp. Raw reads were filtered using FASTP (version 0.18.0). Clean reads were mapped to the *Porphyromonas gingivalis* W83 genome (NCBI RefSeq assembly GCF_000007585.1) using Bowtie2 (version 2.2.8), and reads mapped to ribosome RNA were removed. The edgeR package was used to identify differentially expressed genes (DEGs) among samples with a fold change ≥ 2 and an FDR < 0.05. DEGs were used to conduct the analysis of GO and KEGG, and an FDR < 0.05 was used as the threshold.

The RNA-seq reads have been deposited in the NCBI Sequence Read Archive (SRA) and are accessible with BioProject ID PRJNA1079648.

### 2.8. Statistical Analysis

All experiments were repeated at least 3 times and values are reported as Means ± SD. Data analysis was performed with OriginPro 2018 (OriginLab Corporation, Northampton, MA, USA). Statistical significance was determined by one-way ANOVA and a Duncan test. *p* values less than 0.05 were considered to indicate statistically significant differences.

## 3. Results and Discussion

### 3.1. The Effects of the Flavonoids on Biofilm Quantity Formed by Single or Mixed Strains

**Figure 2:**
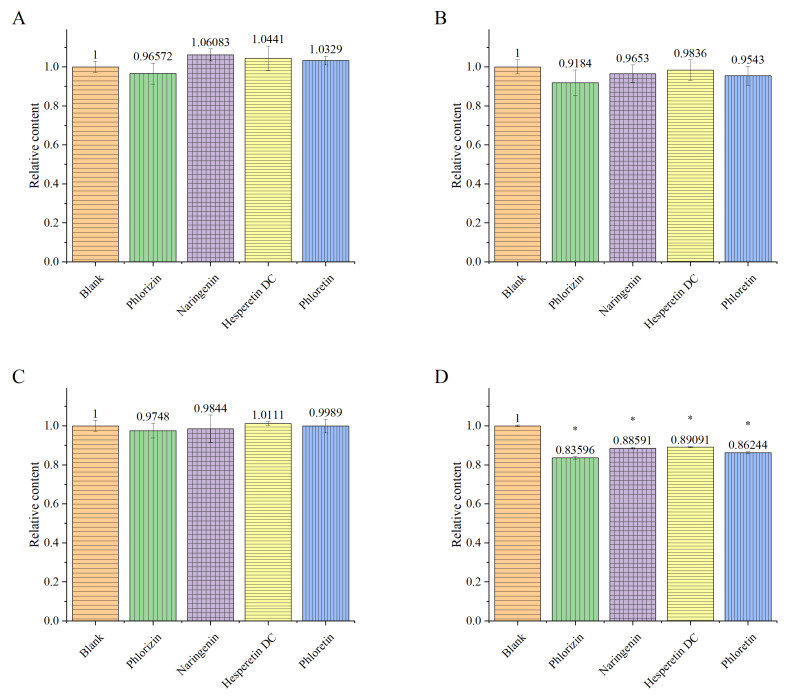
Biofilm contents of *P. gingivalis* (**A**), *F. nucleatum* (**B**), and *S. mitis* (**C**) after treatment, and biofilm content of *P. gingivalis*/*F. nucleatum*/*S. mitis* mixed culture group (**D**). The data were normalized based on the blank group. The values are reported as Means ± SD, and the * indicates that *p* < 0.05, and the data are significant compared with the blank group.

Crystal violet has the capability to stain negatively charged surface molecules and the extracellular matrix of polysaccharides, providing advantages in measuring the quantity of biofilms [34]. The quantitative assessment of the biofilm in the *P. gingivalis* group revealed that phlorizin, naringenin, hesperetin dihydrochalcone, and phloretin had no significant effects on the total content of the *P. gingivalis* biofilm (Figure 2). Among them, only phlorizin exhibited weak inhibitory ability, while the other groups even showed a slight promotion of biofilm formation.

In the *F. nucleatum* group, each group demonstrated a weak inhibitory effect, with phlorizin performing slightly better. For the *S. mitis* group, the candidate molecules had little effect on the biofilm content. When compared with the single-bacterial group, the mixed-bacterial group exhibited a more pronounced inhibitory effect. The total biofilm amount in the four treatment groups significantly decreased, indicating that the candidate molecules exerted a substantial inhibitory effect on the complex biofilm composed of *P. gingivalis*, *F. nucleatum*, and *S. mitis*. This suggests that the candidate molecules had a more significant impact on the mixed-bacterial group than on the single-bacterial group. A complex biofilm is important for the growth, survival, and pathogenesis of *P. gingivalis*, and candidate molecules may play an indirect role in inhibiting the function of *P. gingivalis* biofilms. Quantification experiments cannot reflect whether the structure of the biofilm was destroyed, necessitating further microscopic examination.

### 3.2. The Effects of Flavonoids on Biofilm Metabolic Viability

**Figure 3:**
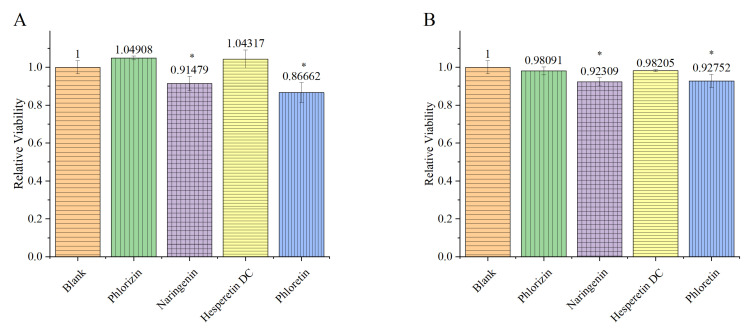
Biofilm viability of *P. gingivalis* (**A**) and *P. gingivalis*/*F. nucleatum*/*S. mitis* mixed culture group (**B**) after treatment. The data were normalized based on the blank group. The values are reported as Means ± SD, and the * indicates that *p* < 0.05, and the data are significant compared with the blank group.

The metabolism of biological tissue can convert resazurin from blue to red. The faster the metabolism, the more rapid the discoloration, and it can be quantitatively detected within a specific wavelength range. According to the monitoring results, in the *P. gingivalis* single-strain group, naringenin and phloretin significantly reduced the metabolic viability of the biofilm, while phlorizin and hesperetin dihydrochalcone showed no effect (Figure 3). This conclusion remains consistent in the *P. gingivalis*/*F. nucleatum*/*S. mitis* complex group, composed of three bacteria, although the inhibitory ability of phloretin was slightly reduced.

Different from the measurement results of the total amount of biofilm, phloretin and naringenin showed significant effects in reducing biofilm metabolic viability to 92.8% and 92.3%, respectively (Figure 3B). Evidently, in comparison with phloretin, the presence of glycosides causes phlorizin to lose its ability to inhibit the metabolic activity of biofilms, and the presence of methoxy groups leads to hesperetin dihydrochalcone losing this ability. The difference in ring closing between naringenin and phloretin did not significantly affect their ability to inhibit the metabolic activity of biofilms. Furthermore, the ability of both naringenin and phloretin to inhibit the metabolic activity of biofilms was established in both the single-bacterial group and the complex group, with no significant difference noted. This suggests that they may possess a broad-spectrum ability to inhibit the metabolic activity of biofilms. The specific mechanism of this action requires further experiments to clarify.

### 3.3. The Effects of the Flavonoids on AI-2 Activity of P. gingivalis

Quorum sensing is an intercellular communication system in which bacteria produce and respond to signaling molecules. Among them, autoinducer-2 (AI-2, furanosyl borate diester) is used for intra- and inter-species communication in Gram-negative bacteria. It is also an important communication system for *P. gingivalis*, regulating its population density, biofilm formation, and multi-strain coaggregation. Several studies have reported that flavonoids can regulate bacterial intercellular communication by inhibiting the activity of AI-2 [35]. Phloretin has also been observed to interfere with the AI-2 communication system of bacteria such as *Listeria monocytogenes* and *Pectobacterium brasiliense* [36,37]. In an antibacterial test against *Streptococcus pyogenes*, phloretin was reported to have the potential to inhibit LuxS protein, which is closely related to the synthesis of AI-2 [38]. The detection of AI-2 activity helps to understand whether candidate flavonoids have the ability to regulate quorum sensing, thereby affecting biofilm formation and cell coaggregation.

**Figure 4:**
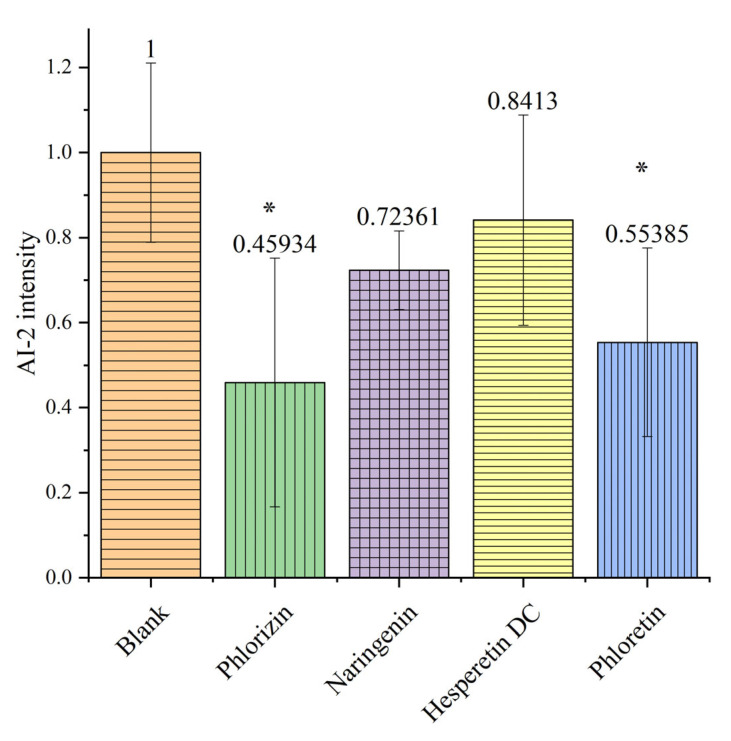
The AI-2 intensity of *P. gingivalis* after treatment in all groups. The data were normalized based on the blank group. The values are reported as Means ± SD, and the * indicates that *p* < 0.05, and the data are significant compared with the blank group.

Based on the detection of chemiluminescence in *V. harveyi*, it was found that treatment with phlorizin and phloretin significantly reduced the activity of AI-2 to 45.9% and 55.4%, respectively (Figure 4). This means that phlorizin and phloretin have a significant ability to interfere with *P. gingivalis*’ intercellular communication. Further verification of the expression of AI-2-related genes is needed to determine whether the mechanism is direct inhibition or due to a decrease in the expression of AI-2.

However, the influence of naringenin and hesperetin dihydrochalcone, which are similar to phloretin in structure, is not as strong as that of phlorizin and phloretin. It is indicated that the structural commonality, the dihydrochalcone backbone, between phlorizin and phloretin is the key to their influence on the AI-2-based quorum sensing of *P. gingivalis*. The closed C-ring of naringenin and the difference in the B-ring of hesperetin dihydrochalcone both reduce their ability to inhibit AI-2 activity.

Naringenin has also been reported to inhibit biofilm formation and LuxS expression in *Streptococcus mutans* [39]. The interference of naringenin with mixed-bacterial biofilms in Figure 2D may include its effects on *streptococci*. Therefore, it can be speculated that the effect of phloretin on mixed-bacterial biofilms may partly come from the interference with the communication between the three bacteria. The fact that phlorizin played a role in the communication of *P. gingivalis* means that the structural difference between phloretin and phlorizin led to a significant decrease in its ability to interfere with the communication between the three bacteria.

### 3.4. Effects of the Flavonoids on Morphology of Biofilms of P. gingivalis

**Figure 5:**
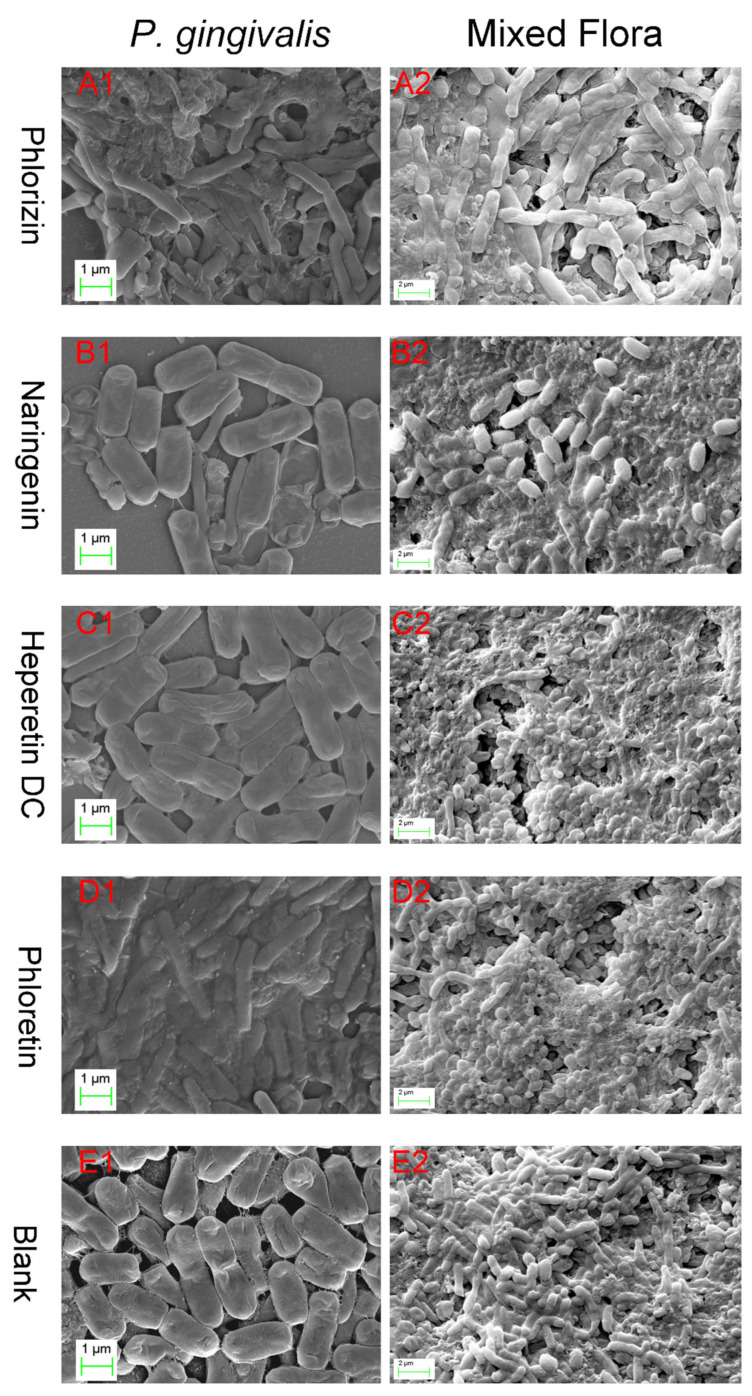
SEM images of *P. gingivalis* single strain and mixed flora (composed of *P. gingivalis*, *F. nucleatum*, and *S. mitis*) after being treated with phlorizin (**A1**,**A2**), naringenin (**B1**,**B2**), hesperetin dihydrochalcone (**C1**,**C2**), and phloretin (**D1**,**D2**); (**E1**,**E2**) are blank groups.

The SEM photos shown in Figure 5 depict the cell morphology and biofilm structure of each group. The phlorizin group (Figure 5A1) and phloretin group (Figure 5D1) exhibited unique and pronounced morphological changes in bacteria, and displayed obvious abnormal aggregation, indicating their special antibacterial mechanisms. In the naringenin group (Figure 5B1) and hesperetin dihydrochalcone group (Figure 5C1), the biofilms appeared broken and scattered, with bacteria detached from the biofilm. Notably, no formed biofilm fragments were observed in the naringenin and hesperidin dihydrochalcone groups. In contrast, the phlorizin group (Figure 5A1) and phloretin group (Figure 5D1) exhibited intact detached biofilm fragments. The shrinkage of bacterial cells and leakage of contents were observed in the naringenin group and hesperetin dihydrochalcone group, confirming their reported antibacterial abilities.

These four candidate molecules showed antibacterial ability against *P. gingivalis* and had different mechanisms of action. From the SEM results, we can see that phlorizin and phloretin have similar effects in changing bacterial morphology, while naringenin and hesperetin dihydrochalcone are relatively similar. The treatments with phlorizin and phloretin caused significant denaturation of the biofilm, and the effect of phloretin was stronger. The effects of naringenin and hesperetin dihydrochalcone may focus on anti-adhesion, causing bacteria to detach from biofilms and hindering the formation of biofilms.

The differences in the SEM images of these four molecules indicate the importance of the common molecular structures of phlorizin and phloretin in enabling their anti-biofilm ability. Additional glycosides retain this anti-biofilm ability, but C-ring closure and methoxy groups destroy this function. This is consistent with the detection results of AI-2 communications. This difference provides valuable guidance for enhancing performance against *P. gingivalis* through molecular modification.

### 3.5. Effects of Flavonoids on Structure of Biofilms Formed by Mixed Strains

*F. nucleatum* and *S. mitis* exert a supportive effect on *P. gingivalis*, resulting in a stronger and more stable biofilm than the one formed by *P. gingivalis* alone. In the mixed-bacterial culture, stable and distinct biofilm structures were observed in all groups (Figure 5). The biofilm structure of the blank group appeared complete with a sense of hierarchy, and the three types of bacteria were balanced and evenly distributed. In contrast, the treatment groups showed significant differences. However, the specific mechanisms varied among the groups.

Treatment with phlorizin caused the biofilm of the mixed-bacterial culture to exhibit a structure resembling melting and then condensation (Figure 5A2). In contrast to the clear layering observed in the other groups, the biofilm in the phlorizin group showed significant deterioration, with many shriveled bacilli displaying leaked contents. *S. mitis* bacteria remained intact but were fewer in number, bound by denatured biofilms. This suggests that phlorizin has a potent destructive effect on mixed-bacterial cultures, not only killing bacteria but also denaturing the biofilm, preventing it from functioning normally.

In the naringenin group, the biofilm exhibited poor layering, and some areas showed denaturation due to the leakage of bacterial contents (Figure 5B2). The bacterial composition revealed that usually, only *P. gingivalis* adhered to the biofilm, with the rare presence of *F. nucleatum* and *S. mitis* wrapped in the biofilm. This indicates that naringenin significantly interferes with the adhesion ability of *F. nucleatum* to mixed biofilms but has little effect on the adhesion ability of *P. gingivalis*.

In the hesperetin dihydrochalcone group, the biofilm structure was complete and layered, but *P. gingivalis* was hardly observed on the biofilm, and the number of *F. nucleatum* was very small. *S. mitis* was more abundant but slightly shriveled, demonstrating bacteriostatic ability (Figure 5C2). Hesperetin dihydrochalcone likely has a strong inhibitory effect on the adhesion ability of the two bacilli but has no obvious effect on the adhesion ability of *S. mitis*. Additionally, hesperetin dihydrochalcone exhibited no apparent destructive effect on the biofilm structure of mixed-bacterial cultures.

The biofilm structure of the phloretin group was complete and layered, with *F. nucleatum* and *S. mitis* more abundant, while *P. gingivalis* was less abundant (Figure 5D2). Compared with the blank group, the numbers of both bacilli were smaller, indicating that phloretin interfered with their adhesion ability. Furthermore, the interference of phloretin with *P. gingivalis* was significantly stronger. The abnormal behavior of the three bacteria in coaggregation confirms the speculation in Section 3.3 that candidate flavonoids interfere with intercellular communication. This indicates that the symbiotic relationship among the three bacteria has been disrupted, which is of great value in solving problems related to *P. gingivalis*.

In summary, structural differences play a crucial role in influencing various aspects of phloretin’s performance. The presence of added glycosides significantly enhances the destructive power against bacterial morphological structures and biofilms. Although biofilm viability is not significantly reduced, the adhesion of other bacteria is greatly affected. A closed ring in the structure weakens the inhibitory effect on *P. gingivalis* but enhances the inhibitory effect on *F. nucleatum* and *S. mitis*, indicating a shift in the target of the candidate molecule. The presence of methoxy groups seems to enhance the anti-adhesion ability to *P. gingivalis* and *F. nucleatum* but fails to produce obvious damaging effects on the complex biofilm. The naringenin group is significantly different from the other three molecules, indicating that the opening of the C-ring plays an important role in maintaining its inhibitory ability against *P. gingivalis*. These diverse changes necessitate caution in improving inhibitory performance against *P. gingivalis* and oral mixed flora through molecular modification, as the mechanism of action and targets of action may undergo alterations.

### 3.6. Analysis of the Effects of Flavonoids on Gene Expression of P. gingivalis Biofilms by Transcriptome Analysis

Extracellular polymeric substances (EPSs) produced by microorganisms are an important basis for the formation of a biofilm matrix [40]. They are mainly composed of polysaccharides, proteins, nucleic acids, lipids, etc. EPS production and biofilm formation are regulated by quorum-sensing mechanisms, which enable bacteria to achieve intercellular communication, affecting population density changes and specific gene expression [41]. This mechanism is particularly important when multiple strains coexist. If the candidate flavonoids affect genes related to biofilm formation, changes may be detected in the synthesis of EPS components or in pathways related to quorum sensing.

**Figure 6:**
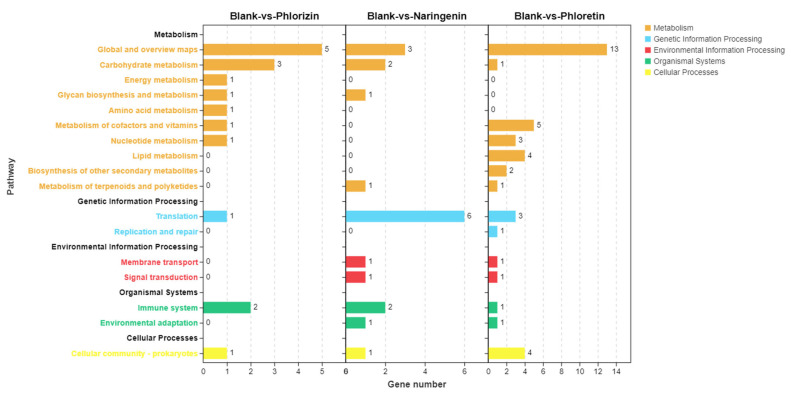
The KEGG pathway enrichment map after phlorizin, naringenin, and phloretin treatment.

Due to the significant differences in the effects of phlorizin, naringenin, and phloretin, these three candidate molecules were selected for transcriptome analysis to further explore their underlying mechanisms. RNA samples were extracted for sequencing comparison, and the mapping ratio of all samples was above 98%, ensuring reliable reference values. The principal component analysis (PCA) results demonstrated substantial differences in functional genes among each group, and the up- and downregulation of differential genes between groups are depicted in Figure 6.

Based on the GO term classification statistics of differentially upregulated and downregulated genes, it was observed that expression differences were mainly enriched in cellular processes, metabolic processes, cellular anatomical entities, catalytic activity, and binding and transcription regulator activity. The GO enrichment results indicated that in all treatment groups, the ribosome was highly activated, highlighting the activation of protein transcription function by the candidate molecules, and the influence of naringenin was greater than that of phlorizin and phloretin. The enrichment analysis results based on KEGG revealed that phloretin interferes considerably with various cellular processes and functions, including signal transduction, translation, membrane transport, lipid metabolism, etc. In comparison, naringenin showed a higher degree of enrichment in translation and outer membrane proteins, with less influence in other aspects compared to phloretin. The influence of phlorizin was concentrated on metabolic pathways.

Further analysis of enriched fatty acid synthesis and metabolic pathways showed that the expression of FabG, FabF, and 3-oxoacyl-[acyl-carrier protein] reductase increased. These genes are related to fatty acid synthesis and are involved in the key steps of the fatty acid biosynthesis elongation cycle [42]. Their upregulation is related to the composition of the cell membrane and biofilm, indicating that bacteria maintain their stability and normal physiological functions by regulating fatty acid synthesis [43]. Therefore, their upregulation may be due to the need to repair cell membranes or biofilm formation after damage. Notably, only phloretin exhibited strong regulation of lipid metabolism.

It is worth mentioning that the biofilm formation function based on the ko02026 pathway was downregulated by all three candidate molecules. The proteins corresponding to this pathway are GlgA and starch synthase, but the change amplitude of the pathway did not meet the significance requirement (*p*.phlorizin = 0.079494, *p*.naringenin = 0.117221, *p*.phloretin = 0.197418), indicating that a higher dose may be needed to enhance their effectiveness. Relatively speaking, phlorizin and naringenin showed a stronger influence on the biofilm formation pathway. The expression of LuxS-related genes did not show significant changes, indicating that the ability of candidate flavonoids to inhibit AI-2 does not come from the regulation of LuxS expression, but may come from the inhibition of LuxS protein function or interference with the action of AI-2.

In the phloretin group, significant changes in the quorum-sensing pathway (ko02024) were detected. The expression of bapA protein in this pathway was significantly reduced, which is related to the synthesis of large surface proteins and participates in biofilm formation. At the same time, changes in the ko03070 pathway showed that the expression of outer membrane proteins was also slightly inhibited. This means that phloretin may have the ability to affect intercellular communication, helping to disrupt the cooperation between *P. gingivalis* and other bacteria.

The expression of AI-2-related genes (such as LuxS) in all treatment groups did not show significant differences. This is in contrast to the detection results of AI-2 levels, which means that the interference of candidate molecules with AI-2 levels is not based on the regulation of related transcription genes, indicating its underlying mechanism.

Overall, according to the GO and KEGG enrichment results, the expression of genes related to biofilm formation was affected by the candidate flavonoids, indicating their role in inhibiting biofilm function. However, the effect of candidate flavonoids in this regard did not reach the significance threshold, suggesting that higher doses may be needed to enhance their effect. Phloretin has the strongest ability to regulate the expression of multiple genes in cells, especially affecting lipid metabolism and quorum sensing. Followed by naringenin, the activation of ribosome function means that the bacterial demand for protein secretion increases. The effect of phlorizin on regulating gene expression is much weaker than that of naringenin and phloretin, and it affects fewer gene pathways, but it shows a stronger influence on inhibiting biofilm formation pathways. Therefore, the inhibitory effects of candidate flavonoids on biofilm formation and adhesion may be mainly based on interfering with biofilm formation and affecting intercellular communication. The direct antibacterial ability of candidate flavonoids may also work from another perspective, cooperating with effects on gene expression to achieve stronger anti-adhesion effects. This conclusion is consistent with the results of biofilm quantification, viability testing, and SEM.

From the perspective of molecular structure, the functional differences in the three candidate flavonoids are obvious. Phloretin has the broadest influence, but phlorizin greatly reduces this influence due to the presence of external glycosides. Although the closed-ring structure of naringenin also reduces its influence, it leads to a stronger effect on its translation function.

## 4. Conclusions

This research reported, for the first time, the inhibition capability of phloretin and its structural analogs on *P. gingivalis* and the mixed flora of *P. gingivalis*/*F. nucleatum*/*S. mitis* in the oral cavity. Due to the different molecular structures of these flavonoids, they exhibit significant differences in their inhibitory effects on the bacteria, and phloretin was shown to be the best choice against *P. gingivalis*. The tested flavonoids significantly affected biofilm formation by the mixed-bacterial culture. Regarding their effects on the mixed biofilm structure, phlorizin had a noticeable destructive effect. Biofilm activity detection showed that both naringenin and phloretin inhibited the metabolic viability of biofilms, and this inhibitory ability performed well in both the single-bacterial group and the complex group. The AI-2 detection results showed that phlorizin and phloretin can affect intercellular communication based on AI-2. The transcriptome results showed that the effect of flavonoids on biofilms mainly focused on interfering with their formation, and phloretin significantly interfered with quorum sensing.

In summary, the structural similarities between phlorizin and phloretin contribute to their powerful anti-biofilm ability against *P. gingivalis*. The addition of methoxy groups may reduce these flavonoids’ anti-adhesion ability against *P. gingivalis*, while a closed loop causes their molecular influence target to shift to affecting *F. nucleatum* and *S. mitis*. These structural modifications may reduce their direct antibacterial ability against *P. gingivalis*. Therefore, specific target objects should be considered, and the structure should be modified in a targeted manner to improve inhibitory performance, or a strategy of using multiple molecules in combination should be adopted.

## Data Availability

The original contributions presented in the study are included in the article, further inquiries can be directed to the corresponding authors.
